# Distinct clusters of movement entropy in children’s exploration of a virtual reality balance beam

**DOI:** 10.3389/fpsyg.2023.1227469

**Published:** 2023-10-17

**Authors:** Håvard Lorås, Ellen Beate Hansen Sandseter, Ole Johan Sando, Lise Storli

**Affiliations:** ^1^Department of Teacher Education, Faculty of Social and Educational Sciences, NTNU, Trondheim, Norway; ^2^Department of Physical Education and Health, Queen Maud University College of Early Childhood Education, Trondheim, Norway

**Keywords:** motor competence, assessment, variability, nonlinear, gross movements

## Abstract

Although assessing motor competence is vital to advancing current understandings of motor development and its significance in various fields, no consensus exists on how the construct should be operationalised and measured. Existing approaches to assessing motor competence in children typically involve applying qualitative and/or quantitative scoring procedures in which children’s performance is evaluated according to certain levels of assessment-specific task performance dependent upon predefined sets of instructions and procedures. Building upon ecological dynamics as a framework, different levels of motor competence can be identified in children’s attempts to coordinate their degrees of freedom while trying to complete the interactive task and environmental constraints. Given the dynamic, nonlinear features of that coordinating process, assessments need to consider the inherit structure of inter- and intra-individual variability in patterns of movement. Against that background, we investigated 7–10-year-old children’s (*n* = 58) whole-body joint kinematics as they freely explored a balance beam in a virtual reality playground. Specifically, we used exploratory cluster analysis to examine the discriminatory capability of utilising joint-specific sample entropy as a window into individual differences in movement coordination that emerged from children’s exploration of the constraints embedded in the virtual task. Among the results, three clusters of children with distinct profiles of movement variability emerged, all of which showed heterogeneous levels of repeatability in joint movements in combination with the level of spatiotemporal exploration on the balance beam that could not be explained by between-cluster differences in age and gender distributions. Those findings suggest that entropy from whole-body movements can be used to cluster children into distinct groups with different profiles regarding the structure of movement variability, which can inform new understandings and the development of gross motor competence assessments for children.

## 1. Introduction

There have been many efforts in recent decades to describe, understand, and assess gross motor competence in children (Barnett et al., 2020). In children, the term *gross motor competence* refers to the level at which children display fundamental motor skills, which are basic gross movements used in activities of daily living and physically demanding pursuits across the human lifespan (Barnett et al., 2016; Logan et al., 2018). Gross motor competence is typically operationalised in three interrelated constructs: locomotion (e.g., running, hopping, jumping, and sliding), object control (e.g., kicking, throwing, and catching), and adjusting and maintaining balance. Using those (and related) constructs, a plethora of test batteries have been developed to assess the level of children’s motor performance (Hulteen et al., 2015, 2020a); those assessments can be broadly divided into product-oriented assessments, which focus on the quantitative outcomes of movement-based tasks, or process-oriented assessments, which consider the qualitative characteristics of how movements are performed according to specific criteria (Logan et al., 2017; Hulteen et al., 2020b; Palmer et al., 2021). Nevertheless, the further development of motor competence assessments has been called for, especially assessments that consider the dynamic, multidimensional nature of motor development that occurs throughout childhood and adolescence, as well as the many task-oriented, individual, and environmental constraints that together influence children’s gross motor competence (Rudd et al., 2016; Hulteen et al., 2022).

The sensitivity and discriminatory capabilities of assessments to detect different levels of gross motor competence are highly dependent upon the operationalisation of task performance. Quantitative scoring procedures, which rank among the most widely used assessment batteries (Kiphard and Schilling, 1974; Bruininks and Bruininks, 1978; Ulrich and Sanford, 1985; Henderson et al., 1992), typically involve measuring temporospatial data concerning the outcomes of children’s movements, including time to complete a task, distance moved, accuracy of movement, and/or successful attempts. Qualitative scoring procedures, by contrast, involve rating how a given task is executed according to criteria for different performance-related components and might be dichotomous in nature—for instance, use “1” and “0” to mean “present” and “absent,” respectively. Independent of the type of scoring procedure, children’s level of motor competence is evaluated against certain assessment-specific levels of task performance that depend upon predefined instructions and procedures.

Such operational definitions of what constitutes skilled or competent movement performance in children appear to be rooted in cognitive theories and models of motor behaviour (Schmidt, 1975; Magill and Hall, 1990; Summers and Anson, 2009). Assessing the potential alignment of a child’s movements with a predefined performance template assumes that some ideal form of movement (e.g., skill) exists for a specific task. In that view, task performance thus depends upon children’s capacity to understand the task and the instructions for it, to process and extract relevant information for planning the task’s execution, and to execute the movements necessary to complete the task (Fitts and Posner, 1967; Henderson and Sugden, 2007). That conceptualisation converges with assessments of whether children have developed cognitive representations that can be matched to meet the given task’s criteria as well as serve as an internal reference of accuracy. Such internal representations provide the structural basis for generating invariant response movements common to a certain class of fundamental movements (i.e., balance, locomotion, and object control) by adjusting and scaling parameters of basic movements (Schmidt, 2003; Keetch et al., 2005). From that perspective, variability in task performance is generally considered to indicate deviation from skilled performance. For instance, if a child demonstrates movements not included in the intended task template or display variations in outcomes on tasks, such movements are essentially treated as unwarranted noise in the data, which inevitably indicates relatively poor motor competence. In cognitively oriented models, those types of intra-individual variability might signify the outcome of poorly processing information and the less skilful execution of task-appropriate movements (Schmidt, 2003; Summers and Anson, 2009; Schmidt et al., 2018).

As an alternative approach to and rationale for operationalising gross motor competence, the ecological dynamics framework provides an integrated explanation for children’s motor competence. Therein, observable movements are viewed as emerging from a self-organising relationship formed between the child, the gross motor task at hand, and the environment in which the assessment is performed (Davids et al., 1994). By extension, intentional motor actions are understood as functional movement solutions that emerge as each child continuously interacts and works within the array of constraints related to the task and environment (Handford et al., 1997). The ecological dynamics framework combines several theories applied in studying motor learning and development, including dynamical systems theory (Thelen and Smith, 1994; Kelso, 1995), complexity sciences (Edelman, 1987), and ecological psychology (Gibson, 1979; Warren, 2006).

The ecological dynamics framework recognises the longstanding knowledge that human motor systems consist of an extraordinary number of independent biomechanical and neuroanatomical properties (e.g., bones, joints, muscles, and neurons), or degrees of freedom, which can be combined in countless ways (Bernshteĭn, 1967). Furthermore, there is abundant evidence of how the coordination of degrees of freedom emerges through self-organisation in complex neurobiological systems (i.e., between muscles, joints, and limbs) during motor development, learning, and performance (Thelen et al., 1987; Mayer-Kress et al., 2009; Kelso, 2022). Self-organisation in coordinating movements is a process in which patterns of movement at the global level (i.e., overall motor behaviour in a task) emerges from the multiple interactions of lower-level components of the motor system (i.e., degrees of freedom). The interaction between degrees of freedom in the motor system, in being nonlinear, dynamic, and continual, provides adaptability and flexibility in the face of changing constraints. Coordinated patterns of movement are emergent properties of those interactions and cannot be understood as the simple addition of individual contributions of lower-level degrees of freedom (Sneyd et al., 2001). All the cited investigations have verified that children’s motor performance, as the outcome of human systems of movement, can be modelled and understood as complex dynamic systems in which functional patterns of motor behaviour emerge in specific contexts.

The natural tendency for self-organisation as it applies to coordinating human movements suggests that children’s motor competence in each task is an emergent property of an attempt to control individual degrees of freedom and work within the interacting constraints and, as such, provides enormous variation in children’s choice of and control over their movements (Chow et al., 2011). The level at which that control is achieved and/or the corresponding movement outcome is thus interconnected to defining features of gross motor competence. As a result, self-organisation in coordinating movement can promote relatively stable patterns of movements as solutions in response to the demands of tasks, especially in conditions with relatively little complexity (Sneyd et al., 2001). In such assessments, children can readily be observed to form a precise pattern of movements, especially if instructions are provided (e.g., as a blueprint, recipe, or specific guideline); however, it is less obvious how a definite pattern of movement can be produced in the absence of such instructions. In terms of dynamical systems theory, those instructions, as task constraints, might heavily influence the use of specific patterns (or states) of coordination that children display during assessment (Kelso and Schöner, 1988; Newell and Ranganathan, 2010). By adjusting important task and environmental constraints, however, essential information about gross motor competence in terms of the level of adaptability and flexibility in coordination can emerge.

The nonlinear nature of the self-organising process by which children control their degrees of freedom in performing motor tasks highlights that movement variability, or fluctuation, is an integrated feature of children’s motor competence. Indeed, variability is a hallmark of the coordination of human movement (Latash et al., 2002; Hadders-Algra, 2010; Hossner and Zahno, 2022) and is conspicuous in the execution of even highly repetitive, well-rehearsed tasks, including self-preferred gait, in which intra-individual variability can be observed on a step-by-step basis (Hausdorff, 2005; Jordan et al., 2007). That dynamic is captured in the term *repetition without repetition*, meaning that no movement or task is performed with the same exact pattern of coordination (Bernshteĭn, 1967). Movement variability has long been identified not as mere random noise in behavioural data that needs to be filtered out but, on the contrary, to contain important information regarding the dynamic status, in developmental terms, of systems of movement (Gibson, 1988; Davids et al., 2006). Indeed, inter-individual differences in the structural and statistical properties of movement variability have been systematically linked to mechanisms of ageing, developmental disabilities, and neurological disorders and are sensitive to systematic variations in environmental and task constraints (Stergiou and Decker, 2011; Stergiou et al., 2013).

The operationalisation of children’s motor competence through the lens of movement variability also addresses the feature of degeneracy inherit in complex neurobiological systems, which captures the idea that similar movement outcomes can be achieved in many ways using different degrees of freedom (Kello et al., 2007). Such potential relates to the *redundancy* of the motor system, meaning the existence of more elements (i.e., degrees of freedom) than necessary to execute motor tasks, thereby resulting in multiple possible solutions to a given motor problem (Scholz and Schoner, 1999). Children’s coordination of degrees of freedom under the confluence of constraints, at least from the perspective of dynamical systems theory, might display variability up to a certain point (i.e., become unstable) and shift to a more stable pattern of movement with less variability. That potential transition between states of behaviour implies that a persistent lack of movement variability may indicate rigid, inflexible motor behaviours with limited adaptability to changing tasks and/or environmental demands (i.e., lower motor competence). Higher movement variability, in that view, might therefore signify the development of a richer repertoire of behaviours and indicate higher motor competence (Stergiou and Decker, 2011; Bisi et al., 2019).

Entropy has been identified as a nonlinear measure and theoretical concept that captures the structure of variability in human movement. Indeed, in many instances, patterns of movement might display a similar statistical dispersion able to be captured by linear measures but have important differences in the underlying structure of movement variability (Yentes et al., 2013). Entropy analysis has provided important insights into the underlying processes of motor control, especially in postural control and gait, across the lifespan (Torres et al., 2013; Busa and van Emmerik, 2016; Bisi and Stagni, 2018). First introduced in thermodynamics, *entropy* can be defined as the loss of information in a time series (Shannon, 1948; Yentes and Raffalt, 2021). Based on what is known about the current state of a movement time series, entropy quantifies the probability of the next state of the movement system. It can also be regarded as a measure of randomness, for it captures the probability that similar patterns of coordinated behaviour will not be followed by additional similar patterns (Stergiou, 2018). When applied to children’s movements in gross motor assessments, entropy is the amount of (microscopic) variability that the motor system displays within various degrees of freedom in the observable behaviour and thus follows self-organisation’s principle of distribution and the coordination of configurations of degrees of freedom that give rise to children’s performance on each motor task (i.e., macroscopic state). Entropy is thus considered to be fundamental to the understanding of emergent properties in complex neurobiological systems considering motor competence in children, which allows establishing variability in coordinating movements ranging from low entropy with highly predictable, repetitive patterns of movement (i.e., potentially lower gross motor competence) to high entropy consisting of less repetitiveness in patterns of movement (i.e., potentially higher gross motor competence).

**Figure 1:**
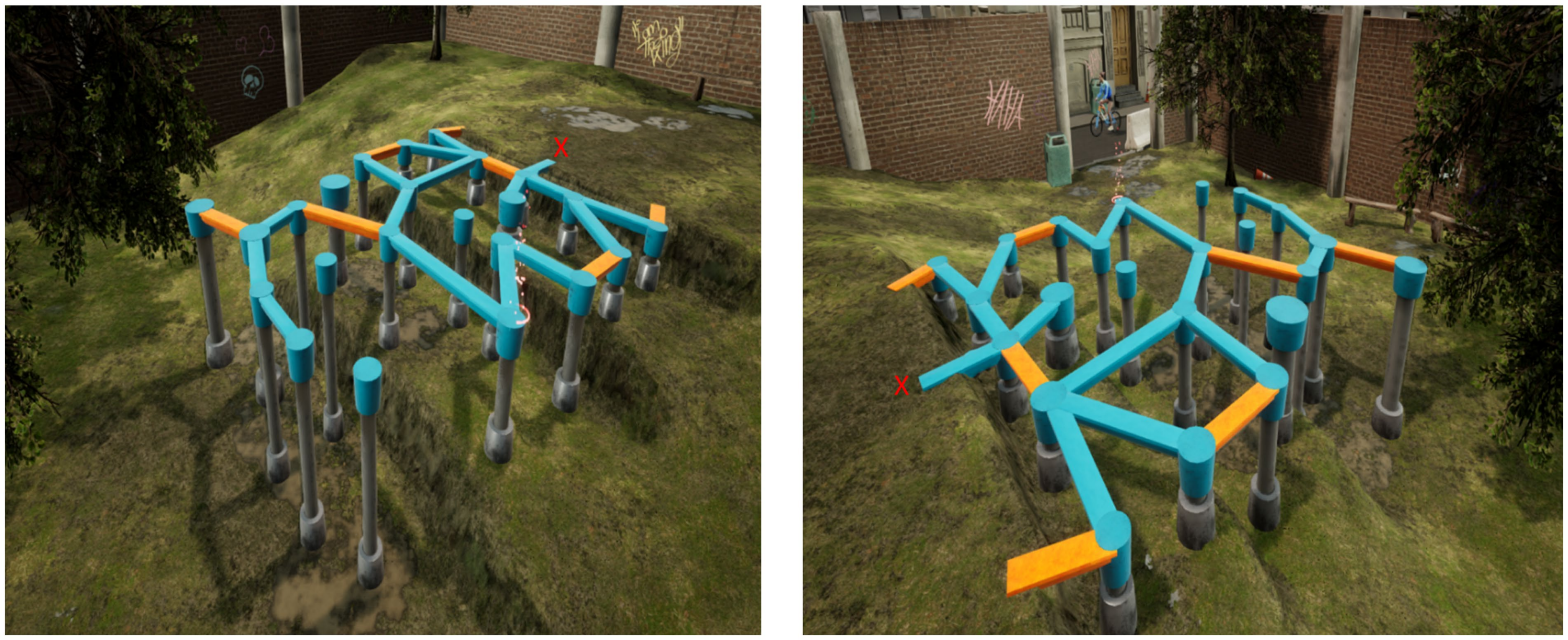
Overview of the virtual reality playground scenario with the balance beam. The red X marks the childrens initial position.

Applying nonlinear measures such as entropy in assessing gross motor competence requires re-examining the design of motor tasks. Such measures require tasks that allow the continuous recording of patterns of movement at sufficient length, which are traditionally examined with relatively low complexity walking and static balancing tasks. E.g., gait patterns are typically assessed by having participants walking a fixed short distance back and forth, and static balance tasks consist of upright quiet standing on a force plate for a fixed period (Yentes et al., 2013; Busa and van Emmerik, 2016; Bisi et al., 2019). These tasks commonly applied to study mechanisms of entropy in human motor control, is also quite like tasks that are a part of widely used assessment batteries for children’s motor competence, such as one-leg (stork) standing for balance assessment and running back and forth a short distance as a part of locomotor assessment (Hulteen et al., 2020a). In the current study, to allow children to freely explore and coordinate their degrees of freedom, we applied a novel balance beam task to be performed on a virtual reality (VR) playground in an urban setting (see Figure 1). In this task, children might potentially integrate various locomotor and static/dynamic balance strategies when they navigate the balance beam. Furthermore, participating children are also provided 3 min of exploration-time, allowing for sampling movement data that can be subjected to entropy analysis.

Emergent motor behaviour on such a VR playground is spontaneous, unpredictable, and self-generated, for there are no specific instructions other than “Explore for yourself, and do not fall off.” Although VR technology is mostly known for simulating environments in gaming or movies, it is increasingly used in different types of health-related research geared towards rehabilitation and physical and/or psychological disabilities (Snider et al., 2010; Ravi et al., 2017; Bortone et al., 2018) and has demonstrated its feasibility in assessments of motor impairments among children. In the latter application, movement strategies have been found to be better assessed in VR scenarios than by using traditional rating scales (Levac et al., 2019). Viewed from an ecological dynamics framework, a realistic balance beam task in VR potentially allows for taking into account the interacting motor, perceptual, cognitive, and affective features that can have an impact upon an individual child’s handling of task and environmental constraints. E.g., it is well known in the context of sports that relatively young children can extract salient cues relevant for maintaining their dynamic balance (Peker et al., 2021; Duncan et al., 2022). Thus, different environmental situations and children’s perceptions of these environments can alter how they successfully achieve a gross motor task (Golding et al., 2014). In the assessment of gross motor competence, VR can thus be applied to design and standardise dynamic tasks in high-complexity environments that might feel realistic and contain recognisable real-world features previously experienced by children (Hulteen et al., 2022).

Altogether, the conceptual frameworks of ecological dynamics that guided the current study advances that children’s motor competence emerges as children attempt to coordinate their degrees of freedom while at once seeking to work within the constraints of the task and environment. Given the dynamic, nonlinear features of that process of coordination, the assessment of motor competence needs to consider the inherit structure of inter- and intra-individual variability in children’s emergent patterns of movement. Against that backdrop, the objective of the current study was to investigate whether measures of whole-body entropy have discriminatory capability through being able to classify individual differences in children’s coordination of movements. Specifically, we performed exploratory cluster analysis to determine whether movement entropy in children’s exploration of constraints embedded in a virtual reality balance beam can be used to cluster children into distinct groups with different profiles regarding the structure of movement variability. Provided the features of entropy as a nonlinear metric, we hypothesised that clusters of children would emerge with profiles of relatively low entropy and repetitive patterns of movement, higher entropy consisting of less repetitiveness in patterns of movement, or a combination of these profiles. Furthermore, it was also hypothesised that cluster differences would emerge in the spatiotemporal measures of the children’s exploration of the VR balance beam.

## 2. Methods

### 2.1. Participants

We invited 76 children 7–10 years old (i.e., in Grades 2–4 in Norwegian schools) attending a primary school in a rural area in Central Norway to participate in our study. The school was selected for convenience sampling given its proximity to our university, which facilitated transportation and the twice-daily installation of equipment on each visit. In the school, parents or guardians for 64 of the children (84%) provided their written informed consent for their children’s participation. However, two of the 64 children (3%) declined their consent; thus, the initial sample consisted of 62 children, all of whom started the VR simulation. Of them, two children failed to complete the test; one experienced virtual reality sickness (cyberkinetosis), while the other thought that the tasks were too complicated. Beyond that, data from two other children were omitted from analysis due to technical malfunctions. The final sample therefore consisted of 58 children (31 boys, 27 girls); 12 were from Grade 2, 23 from Grade 3, and 23 from Grade 4, and their mean (*SD*) age was 8.9 (0.8) years. The Norwegian Social Science Data Services (NSD), part of the Norwegian Agency for Shared Services in Education and Research, approved the project (Project No. 324155), and all data and information related to the project were handled in accordance with the NSD’s ethics guidelines.

### 2.2. Apparatus

The VR equipment (HTC VIVE, New Taipei City, Taiwan) was installed in the school’s gymnasium, and a 7 × 6 m area was established and calibrated for the VR software (Steam VR 2.0) with a Steam VR Base Station located in each corner. Participating children were equipped with the VIVE Pro Eye Headset and five VIVE 3.0 trackers on the right and left wrists, the right and left feet, and another just below the navel. For motion capture, 17 wireless inertial measurement units (IMUs) from the MTw Awinda system (Xsens Technologies, Enschede, the Netherlands) were attached to the children with Velcro straps on the forehead, sternum, palmar side of the right and left hands, the lateral side of the upper right and left arms, the wrist right and left wrists, the lateral side of the lower right and left legs, the lateral side of the right and left thighs, the right and left feet, the upper part of the right and left scapulae, and the lower back (i.e., L5, height of the iliac spine). In general, MTw Awinda samples data at 60 Hz, and the IMUs are 47 × 30 × 13 mm and weigh 16 g. The system provides 3D kinematical data (i.e., position, velocity, acceleration, and orientation) on the movement of 23 body segments as well as the angular movements of 22 joints.

### 2.3. Procedure

Participating children were retrieved one by one from their respective classrooms and escorted to the gymnasium. After being familiarised with the equipment and safety routines, the child entered the VR environment in a warm-up scenario in which they could explore an urban park. In that scenario, necessary calibrations of the motion capture and VR systems were conducted. After 3–5 min of warm-up and familiarisation, the surround-sound headset was turned on for further verbal instructions, and the child entered the first scenario. The first two scenarios were not analysed in the current paper because they are being examined in a larger-scale study titled “Virtual Risk Management – Exploring Effects of Childhood Risk Experiences through Innovative Methods”; for additional specific information regarding the larger-scale study, interested readers are referred to a protocol paper (Sandseter et al., 2023). Children completed each scenario in the same order, which took approximately 15 min. The last 3 min was devoted specifically to the playground scenario that we analyse in the current paper.

### 2.4. The virtual reality balance beam

The playground scenario (see Figure 1) presented a play structure for balancing in an urban playground environment. At the beginning of the scenario, children are provided with in-ear information via a pre-recorded woman’s voice that they are free to move, explore, and play as they like but to try to avoid falling from the structure. If the child fell off, then the voice told the child that they have fallen off, and the software allows the child to return to the starting point and to resume moving around freely. Thus, the scenario did not contain a fixed objective for the children to achieve. In the current sample of children, only 7 (12%) of the children had a virtual fall off the playground. In the VR environment, the children could see their feet displayed as transparent shoes. The virtual balancing structure comprised a complex pattern of balance beams of varying widths, vertical pillars with varying diameters, and four different height zones. As visible in Figure 1, the scenario allows for navigating various paths on the balance beam, which were too narrow to allow for normal gait patterns. It was also possible to jump between isolated beams and pillars, while at the same time considering different heights beneath. The blue and orange colours on the beams were chosen to add some variation in the texture of the balancing structure. The children received 3 min to explore the scenario, while sounds of the urban environment (e.g., cars, bikes, and people) played through the headphones to facilitate the immersive VR experience. About half of the children (56%) reported to have tried any type of virtual reality experience before participating in the current study.

### 2.5. Data analysis

Data files were pre-processed in MTv Awinda software (Xsens, Enschede, the Netherlands) to ensure that all data were transferred and re-transmitted. Raw data were exported and further processed in MATLAB R2022a (MathWorks Inc., Natick, MA, United States) using in-house algorithms. Data selected for processing included the 3D positions of the right and left feet and forearms (i.e., determined segments in the Xsens system), the kinematics of the pelvis (i.e., position, velocity, and acceleration), and joint data from the head and the right and left shoulders, elbows, hips, knees, and ankles. The start and end of the entire playground epoch were first determined in Matlab (i.e., the *n* of frames of total playground epoch was 10,800), and thereafter visually inspected to ensure that the stationary start or stop (i.e., when the children were not moving) of raw signals were removed from further analysis. To preserve variability in the data, no further filtering was applied before entropy analysis (Stergiou, 2018). Sample entropy (SampEN) was thereafter calculated using MATLAB code provided by PhysioNet (Goldberger et al., 2000), based upon algorithms developed by Richman and Moorman (2000). Entropy was defined as the negative logarithm for conditional properties that a series of data points within a certain distance (*m*) would be repeated within the distance *m* + 1. Two parameters thus needed to be set before the calculation of SampEN: (a) the relative tolerance limit (*r*), or the number times the standard deviation (*SD*) of the data, and (b) the vector length (*m*). In our study, *m* was set at 2 and *r* at 0.2 (Yentes et al., 2013; Yentes and Raffalt, 2021). Time series varied in length from 10,000–10,800 frames due to the removal of the mentioned stationary start and stop. SampEn was calculated from the entire movement time series of the following joints bilaterally: ankle dorsiflexion–extension, knee flexion–extension, hip flexion–extension, shoulder abduction–adduction, elbow flexion–extension, and head yaw and pitch. Those joint movements, selected to capture whole-body movements, constituted important degrees of freedom in locomotory behaviours (Assaiante, 1998; Brandes et al., 2006; Da Costa and Rocha, 2013). Furthermore, a modest selection of joint variables was needed for statistical purposes due to our modest sample size. As overall spatiotemporal measures of the children’s exploration of the VR balance beam, the area (i.e., range moved in *x* direction × range moved in *y* direction), distance (i.e., sum of displacement in *x* and *y* directions), mean velocity (i.e., anterior–posterior, and mediolateral), and mean acceleration (i.e., anterio-posterior, and mediolateral) were computed after. Before the computation of these variables, the frequency spectrum content of the raw data was inspected with the periodogram method, and a low pass, zero phase, 1 Hz, 4th order, Butterworth filter was applied. Velocity (m/s^2^) and acceleration (m/s^3^) were measured directly in the MTv Awinda system, and all spatiotemporal measures were obtained from the movements captured by the pelvis segment.

### 2.6. Statistical analysis

Prior to analysis, all variables were tested and confirmed for normality by non-significant Kolmogorov–Smirnov tests and the inspection of histograms and Q–Q plots. Before proceeding to cluster analysis, intercorrelations (Pearson’s *r*) between entropy measures were examined based on the rationale that, in the presence of a strong correlation between variables (*r* ≥ 0.80), the variables are not sufficiently unique to identify distinct clusters and can be overrepresented in the clustering solution. In the remaining data, close inspection of the correlation matrix indicated that the correlations between sample entropy from the right and left knees (*r* = 0.93) and between sample entropy from right and left hips (*r* = 0.87) were at that level. Thus, mean entropy across the right and left knees and across the right and left hips were used in further analysis.

Cluster analyses were conducted to group children’s sample entropy profiles into potential distinct clusters by comparing two hierarchical agglomerative clustering methods: the Ward method and the between-groups average linkage method. These methods were chosen to strike a balance between minimizing the variance within each cluster (Ward method) and minimizing the average dissimilarity between all pairs of data points in merged clusters (average linkage). For both methods, the squared Euclidean distance was the measure of proximity as it comprises the most common dissimilarity metric when working with numeric data (Yim and Ramdeen, 2015). The final number of clusters was determined by examining the agglomeration schedules and dendrograms generated for both techniques. The visual inspection of each cluster’s membership was performed to assess the utility of each potential cluster solution. Once the most statistically robust and theoretically relevant cluster solution was identified, *k*-means iterative partitioning was used to fine-tune the clusters (Eldred and Darrah, 2010; Yim and Ramdeen, 2015). Last, comparative cluster analysis was conducted on demographic data, spatiotemporal linear measures, and SampEN with one-way ANOVAs with the partial eta squared (*η*^2^) applied as the indicator of the effect size, interpreted as small (0.01), medium (0.06), and large (0.14; Richardson, 2011). *Post hoc* pairwise comparisons at the level of the overall main effects were conducted with Bonferroni-corrected paired samples *t* tests with Cohen’s *d* applied as a measure of the effect size, in which 0.2, 0.5, and 0.8 were considered to indicate small, moderate, and large effects, respectively (Lakens, 2013). All statistical calculations were performed in SPSS Predictive Analytics (IBM, Armonk, NY, United States) version 29.0 with α = 0.05 as the criterion for statistical significance.

## 3. Results

### 3.1. Cluster analysis

**Figure 2:**
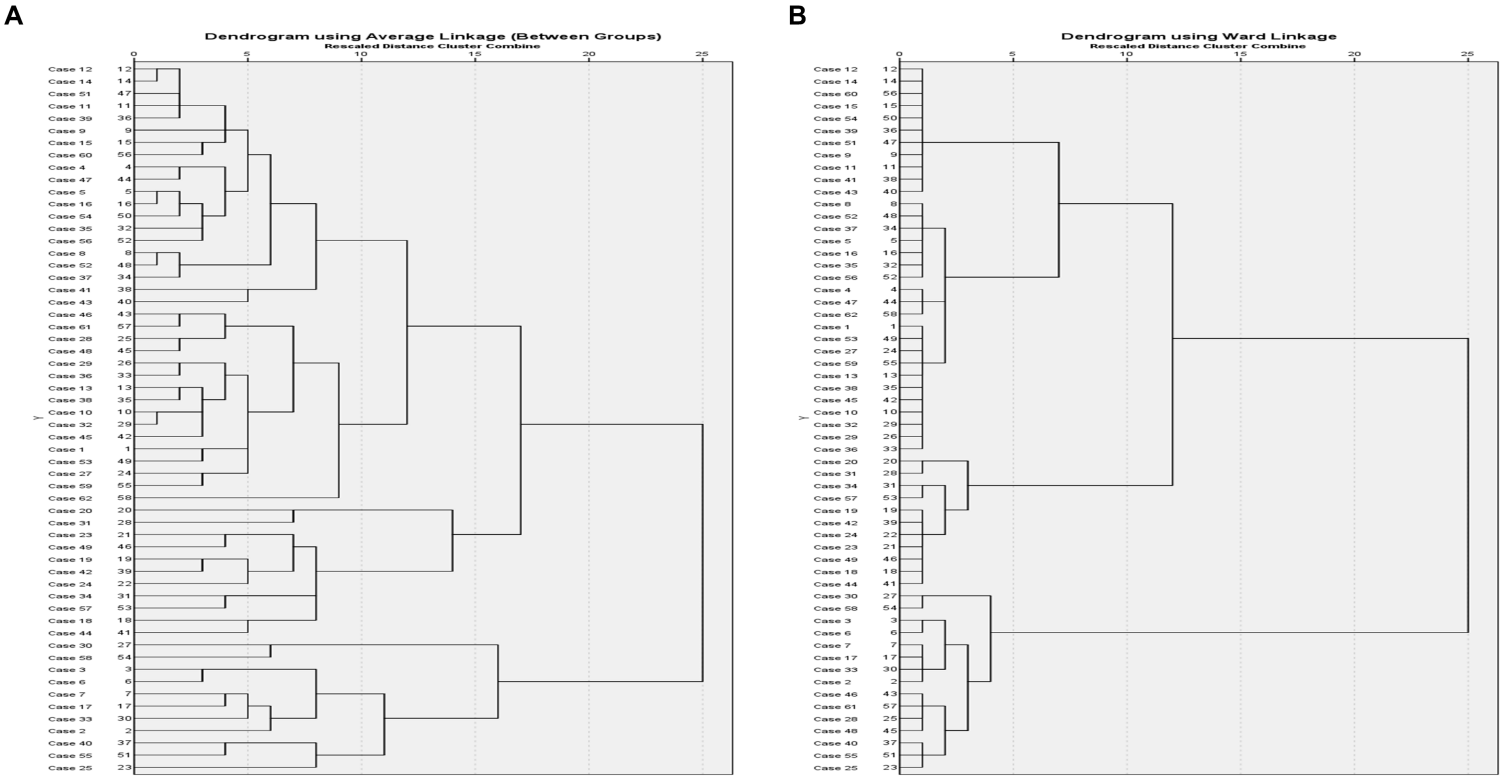
Dendrograms for the average linkage method **(A)** and the ward linkage method **(B)**.

Hierarchical agglomerative clustering with the between-groups average linkage method and the Ward method indicated that the most discrete change in squared Euclidean distance between the adjacent number of clusters appeared between the change in distance between six compared with three clusters. As evident from the dendrogram in Figure 2, a three-cluster pattern represented the point which an abrupt change in the squared Euclidean distance appeared in the agglomeration schedule. Further *k*-means cluster analysis with the iterative partitioning method indicated significant between-cluster differences for all SampEN measures for a three-cluster solution (*F* ≥ 10.64, *df* = 2, *p* < 0.001).

### 3.2. Comparative cluster analysis

Descriptive statistics of the variables studied appear in Table 1. No significant between-cluster differences emerged in age, grade, or number of boys and girls (*p* > 0.05).

**Table 1:** Descriptive statistics of demographics, sample entropy, and spatiotemporal measures across clusters.

					Overall difference
Variable		Custer I (*n* = 15)	Cluster II (*n* = 30)	Cluster III (*n* = 13)	Between clusters
*Demographics*					
Age (years)		9.28 (0.89)	8.72 (0.79)	8.92 (0.71)	*ns*
Girls/boys (*n*)		5/10	13/17	9/4	*ns*
2nd/3rd/4th grade (*n*)		3/3/9	7/14/9	2/6/5	*ns*
*Sample entropy*					
Ankle dorsiflexion-extension	Right	0.32 (0.03)	0.22 (0.04)	0.23 (0.03)	*p* < 0.001
	Left	0.33 (0.04)	0.24 (0.04)	0.25 (0.04)	*p* < 0.001
Knee flexion-extension		0.28 (0.05)	0.19 (0.05)	0.17 (0.04)	*p* < 0.001
Hip flexion-extension		0.25 (0.03)	0.18 (0.03)	0.19 (0.02)	*p* < 0.001
Shoulder abduction-adduction	Right	0.15 (0.03)	0.10 (0.02)	0.14 (0.04)	*p* < 0.001
	Left	0.15 (0.03)	0.09 (0.02)	0.15 (0.04)	*p* < 0.001
Elbow flexion-extension	Right	0.12 (0.05)	0.07 (0.03)	0.18 (0.05)	*p* < 0.001
	Left	0.13 (0.07)	0.07 (0.04)	0.20 (0.06)	*p* < 0.001
Head rotation	Pitch	0.10 (0.02)	0.08 (0.02)	0.07 (0.02)	*p* < 0.001
	Yaw	0.12 (0.03)	0.08 (0.02)	0.09 (0.03)	*p* < 0.001
*Spatiotemporal playground measures*					
Distance (m)		56.58 (15.40)	39.27 (9.21)	38.55 (11.06)	*ns*
Area (m^2^)		24.99 (2.41)	25.91 (7.32)	24.28 (3.97)	*p* < 0.001
Velocity (m/s^2^)	Mediolateral	0.29 (0.05)	0.20 (0.06)	0.22 (0.06)	*p* < 0.001
	Anterior – posterior	0.32 (0.09)	0.22 (0.05)	0.22 (0.06)	*p* < 0.001
Acceleration (m/s^3^)	Mediolateral	0.90 (0.18)	0.68 (0.16)	0.61 (0.15)	*p* < 0.001
	Anterior - posterior	0.93 (0.22)	0.69 (0.14)	0.62 (0.16)	*p* < 0.001

ns: no significant difference between clusters (*p* > 0.05).

#### 3.2.1. Sample entropy

As indicated in Table 1, one-way ANOVAs indicated that all measures of sample entropy significantly differed between clusters for both upper-extremity joints (*F* ≥ 10.66, *df* = 2, *p* < 0.001, *η*^2^ ≥ 0.28) and both lower-extremity joints (*F* ≥ 22.01, *df* = 2, *p* < 0.001, *η*^2^ ≥ 0.44). *Post hoc* pairwise comparisons of clusters for lower-extremity joints indicated that ankle entropy (i.e., right, and left) differed significantly between Clusters I and II and Clusters I and III (*t* = 6.26, *df* = 27, *p* < 0.001, *d =* 2.01), albeit no significant difference was found between Clusters II and III (*t* = 1.42, *df* = 32, *p* = 0.08, *d =* 0.41). Similarly, knee joint entropy (i.e., flexion-extension) significantly differed between Clusters I and II and Clusters I and III (*t* = 6.79, *df* = 25, *p* < 0.001, *d =* 2.52) but not between Clusters II and III (*t* = 1.28, *df* = 25, *p* = 0.11, *d =* 0.41), while hip joint entropy (i.e., flexion-extension) differed significantly between Clusters I and II and Clusters I and III (*t* = 8.32, *df* = 34, *p* < 0.001, *d =* 2.54) but not between Clusters II and III (*t* = 1.29, *df* = 31, *p* = 0.21, *d =* 0.38).

For the upper extremities, shoulder entropy (i.e., right, and left) differed significantly between Cluster I and Cluster II (*t* = 7.20, *df* = 23, *p* < 0.001, *d* = 2.49) and between Clusters II and III (*t* = 5.77, *df* = 15, *p* < 0.001, *d* = 1.92) but not between Clusters I and III (*t* = 0.43, *df* = 25, *p* = 0.41, *d* = 0.11). Elbow entropy (i.e., right, and left) differed significantly between Clusters I and II (*t* = 4.42, *df* = 21, *p* < 0.001, *d* = 1.40), between Clusters I and III (*t* = 3.08, *df* = 26, *p* < 0.001, *d* = 1.16), and between Clusters II and III (*t* = 8.89, *df* = 41, *p* < 0.001, *d* = 2.95). Furthermore, head pitch entropy differed significantly between Clusters I and II (*t* = 3.99, *df* = 26, *p* < 0.001, *d* = 1.31) and between Clusters I and III (*t* = 3.90, *df* = 26, *p* < 0.001, *d* = 1.46) but not between Clusters II and III (*t* = 0.42, *df* = 25, *p* = 0.34, *d* = 0.13). Last, head yaw entropy differed significantly between Clusters I and II (*t* = 5.68, *df* = 22, *p* < 0.001, *d* = 1.99) and between Clusters I and III (*t* = 3.63, *df* = 26, *p* < 0.001, *d* = 1.37) but not between Clusters II and III (*t* = 1.18, *df* = 19, *p* = 0.26, *d* = 0.43).

#### 3.2.2. Spatiotemporal variables

As indicated in Table 1, no significant between-cluster differences emerged in the size of the area explored in the playground scenario (*F* ≥ 0.39, *df* = 2, *p* = 0.068, *η*^2^ ≥ 0.01). For overall distance moved in the 3-min period, overall significant between-cluster differences were found (*F* ≥ 12.98, *df* = 2, *p* < 0.001, *η*^2^ ≥ 0.32). Bonferroni-corrected pairwise comparisons indicated significant differences between Clusters I and II (*t* = 4.01, *df* = 19, *p* < 0.001, *d* = 1.49) and between Clusters I and III (*t* = 3.59, *df* = 25, *p* < 0.001, *d* = 1.33) but not between Clusters II and III (*t* = 0.21, *df* = 20, *p* = 0.84, *d* = 0.07). Furthermore, significant between-cluster differences for mean acceleration (i.e., anterior–posterior and mediolateral) were observed (*F* = 14.48, *df* = 2, *p* < 0.001, *η*^2^ = 0.35). Pairwise comparisons indicated a significant difference between Clusters I and II (*t* = 4.03, *df* = 24, *p* < 0.001, *d* = 1.41) and between Clusters I and III (*t* = 4.56, *df* = 25, *p* < 0.001, *d* = 1.71) but not Clusters II and III (*t* = 1.34, *df* = 23, *p* = 0.08, *d* = 0.52). Similarly, for mean velocity (i.e., anterior–posterior and mediolateral), significant between-cluster differences also emerged (*F* = 14.15, *df* = 2, *p* < 0.001, *η*^2^ = 0.34). Pairwise comparisons indicated a significant difference between Clusters I and II (*t* = 5.41, *df* = 30, *p* < 0.001, *d* = 1.67) and between Clusters I and III (*t* = 3.56, *df* = 25, *p* < 0.001, *d* = 1.41) but not between Clusters II and III (*t* = 0.82, *df* = 23, *p* = 0.21, *d* = 0.27).

## 4. Discussion

The chief aim of our study was to investigate individual differences in movement variability within 7–10-year-old children’s exploration of a balance beam on a VR playground, and whether these individual differences could be classified via whole-body entropy. The discriminatory capability of these measures was examined by performing an exploratory cluster analysis of sample entropy estimates obtained from a 3-min time series of upper-extremity (i.e., head, shoulder, and elbow) and lower-extremity (i.e., ankle, knee, and hip) joint movements, specifically to examine whether entropy from multiple joints could be used to group children in distinct clusters with different profiles of structural movement variability. The cluster analysis indicated the presence of three clusters, each with a distinct profile, with sufficient internal (i.e., within-group) homogeneity and external (i.e., between-group) heterogeneity (see Table 1). Overall, effects for between-cluster comparisons were found for all joint-specific measures of sample entropy and spatiotemporal measures (i.e., distance, velocity, and acceleration) describing children’s exploration of the VR balance beam. Furthermore, no between-cluster differences emerged for demographic variables (i.e., age, grade, and number of boys and girls).

### 4.1. Cluster I: higher overall whole-body entropy and degree of spatiotemporal exploration

Cluster I of children (*n* = 15) was highly different from the other clusters given high between-cluster effect sizes (Cohen’s *d* ≥ 1.4) on higher sample entropy from lower-extremity joints and across spatiotemporal measures (see Table 1). Children in Cluster I thus moved and accelerated faster while exploring the VR scenario, which allowed a greater distance of movement in the 3-min period and at once preserved more joint-specific entropy than for their peers in the sample of typically developing children. Despite the lack of consensus regarding the interpretation of entropy in the coordination of movement (Stergiou and Decker, 2011; Yentes and Raffalt, 2021), higher entropy levels are typically considered to indicate less repetitiveness and different states of variability in functional patterns of movement. Because the children in Cluster I spent more time navigating the VR scenario, they also necessarily spent more time adapting to changing constraints embedded in their choices made throughout the 3-min period.

As shown in Figure 1, the structure of the balance beam seemed to present many types of locomotory and stability challenges, which must be adapted according to where and how children navigate. For example, moving towards the outer layer of the VR scenario (high ground, see Figure 1) ultimately presents the choice of moving farther out to a single pillar or turning and moving back in the direction travelled. In the case of the first choice, a new pattern of movement can emerge when a child moves out to a single pillar; in the latter case, a child turns and potentially repeats a pattern of movement in the opposite direction. Higher entropy in Cluster I can therefore be explained by the emergence of different levels of functional and adaptive variability in the VR scenario, compared with their peers in the sample. For an explanation of between-cluster differences in entropy and overall spatiotemporal exploration, those children might have a richer repertoire of motor strategies that can be adapted and varied according to the specifics of the situation (Hadders-Algra, 2010). Thus, the degree of entropy (i.e., variability) in their control of locomotion and stability allows their dynamic motor system to transition between coordinative states more rapidly (Stergiou and Decker, 2011). In our study, that dynamic laid the foundation for higher average speed and acceleration and more distance covered in the VR scenario. Indeed, higher entropy in coordinative states has been linked to facilitated adaptation towards environmental and task constraints in many different contexts (Cordier et al., 1994; Stergiou, 2018; Becker and Hung, 2020).

### 4.2. Cluster II: lower overall whole-body entropy and less spatiotemporal exploration

Cluster II (*n* = 30) that emerged from the hierarchical agglomerative clustering analysis (see Table 1) appeared to demonstrate the lowest overall whole-body, joint-specific sample entropies in between-cluster comparisons, as well as less variability in spatiotemporal patterns than in Cluster I. Cluster II, the largest group in our sample, appeared to explore the VR scenario with more repetitive joint movements (i.e., lowest overall variability in the sample) and with less speed, acceleration, and, consequently, distance covered than the children in Cluster I. It has previously been shown that younger children (i.e., 6–7 years old) might display less entropy in stride-to-stride variability in simple walking tasks than older children (i.e., 9–10 years old), which has been interpreted as an age-dependency of such nonlinear metrics when capturing locomotory maturation (Bisi and Stagni, 2018; Bisi et al., 2019). In our sample of children, however, we did not observe any significant between-cluster difference in age. That outcome indicates that measuring joint-specific entropy from whole-body movements can reveal individual differences in children’s coordination of movements that is sensitive to other developmental processes beyond what can be ascribed to a less mature motor system, even within cohorts of similarly aged children.

Lower values of sample entropy, as observed in Cluster II, can be interpreted as greater similarity in patterns of movement across the 3-min period and more repetitive, less exploratory gross motor movements (Smith et al., 2017) when navigating the VR balance beam. From the perspective of ecological dynamics (Davids et al., 1994; Seifert et al., 2013; Button et al., 2020), less entropy and spatiotemporal exploration demonstrated by the children in Cluster II indicate a different self-organising process in the dynamic interaction between the child (i.e., individual constraints) and the VR scenario (i.e., environmental and task constraints). As children freely explore and navigate the VR balance beam, there are many opportunities for locomotory and balance-related actions that can be utilised. That process depends upon the affordances emerging in the individual child–VR scenario interactions as a part of ongoing perception–action cycles. Although there are many possibilities for action embedded and afforded in the VR scenario, differences in the utilisation of those affordances might explain the between-cluster differences in entropy and exploration observed in our study. That might occur through several integrated dynamic processes, for lower entropy can provide a less diverse repertoire of movements and impact the perception of affordances, while moving with less entropy impacts the ongoing perception–action cycle. Differences in entropy can also occur because of perceiving and acting upon different affordances. In either case, our results warrant further examination regarding the possibility that nonlinear measures such as entropy can provide an indication of the individual–environment fit (O’Sullivan et al., 2020) that emerges in the assessment of gross motor competence when children are allowed to freely explore tasks.

### 4.3. Cluster III: higher upper-extremity entropy and lower spatiotemporal exploration

Cluster III, indicating the last distinct profile suggested by hierarchical analysis, consisted of children (*n* = 13) with the highest sample entropy obtained from upper-extremity joints and lower spatiotemporal measures obtained from exploring the VR balance beam. Children in Cluster III thus moved more slowly and explored less than children in Cluster I and with more variability in their upper-extremity joints than children in Cluster II. The difference between Clusters II and III indicates the degeneracy and redundancy inherit in motor coordination, as captured by the application of principles derived from perspectives on complex neurobiological systems (Seifert et al., 2016). That is, the two clusters show similarity in spatiotemporal exploration while at the same time achieving that exploration with different structure of movement variability. Moreover, self-organisation in movement coordination, as a nonlinear process, assumes that patterns of movement at the global level (i.e., overall motor behaviour) can emerge from multiple interactions between lower-level components of the motor system, which is not simply an addition of the various components’ contributions (Kelso, 1995; Sneyd et al., 2001).

The explanation as to why higher entropy in upper extremities emerged as a between-cluster difference is not a straightforward one. The nature of the VR balance beam primarily promoted exploration through the movement of the lower extremities, thereby making it highly plausible for between-cluster differences to originate from those sources. At the same time, upper-body movements, especially those of the arms, are known to assist children in maintaining dynamic balance in locomotory tasks (Meyns et al., 2012; Hill et al., 2019), and moving with less regularity in the upper extremities (i.e., higher entropy) can boost flexibility and adaptability in maintaining balance. Indeed, lower entropy in movements involved with maintaining dynamic processes that contribute to the control of task-specific upright standing has been linked to changes in various physiological functions due to age and disease (Busa and van Emmerik, 2016; Anderson and Button, 2017). However, less repeatability in upper body movements can also be interpreted as a sign of excessive and/or redundant movements. From that perspective, it might be hypothesised that higher entropy in the upper extremities, as observed among the children in Cluster III, might emerge due to compensatory arm movements (e.g., to regain balance) that are not necessarily associated with adaptivity provided by the individual motor repertoire. Whether this feature provides an explanation for our differences between cluster II and II, relates to the divergence in interpretating variability and highlights the problematic nature of assessing what is potentially “good variability,” so to speak, versus potentially “bad variability” in evaluating children’s performance using gross motor assessments (Hossner and Zahno, 2022). Viewed through the lens of ecological dynamics, all movements and their outcomes contain variability, and the further development of measures (e.g., sample entropy) for assessing children’s gross motor competence stands to disentangle the relative contribution of functional and non-functional variability in adapting motor repertoires to shifting environmental and task constraints.

### 4.4. Between-cluster differences in entropy and spatiotemporal exploration

Although age did not seem to be a discriminatory factor in our study for between-cluster differences, other demographic variables such as gender have been shown to be associated with the level of gross motor competence. For example, a typical finding is that boys outperform girls on tests associated with more gross movement coordination (Bolger et al., 2021; Gosselin et al., 2021). In our study, we did not observe such gender-based differences, however, because the ratio of boys to girls across clusters was not significantly different (see Table 1). As a novel finding of our study that awaits further research, it can be hypothesised that individual differences in whole-body measures of joint-specific entropy emerging from relatively free exploratory tasks such as the VR balance beam scenario cannot be explained by children’s age (i.e., 7–10 years old in our study) or gender.

The overall finding of heterogenous clusters of children that is based upon their structure of movement variability, as well as in aspects of their overall spatiotemporal behaviour in the virtual balance beam, suggests that novel methods for classifying children’s motor competence can be developed that is based upon a combination of free task exploration and whole-body movement coordination. This provides a conceptually different approach compared to the common taxonomies of motor competence in children where classification to a larger degree is based upon assessment-specific levels of task performance dependent upon predefined instructions and procedures (Newell, 2020). Classification based upon entropy-based metrics in exploratory task paradigms relies to a larger extent on aspects of adaptability and flexibility in children’s motor behaviour (Stergiou and Decker, 2011; Bisi et al., 2019) and might therefore guide the development of *different* motor competence profiles, not just level of motor competence based upon task performances. E.g., children in cluster I, in which demonstrated the highest overall degree of entropy in the current study, might represent a motor competence profile with children capable of changing movement strategies and utilise functional variability (Phillips et al., 2012) that allow for a richer repertoire in handling of various environmental and task constraints. This might be advantageous when children navigate various sport, physical education and/or physical activity contexts, when compared to motor competence profiles of children less able to adapt to, and flexibly explore, various confluence of constraints. It might be speculated that children in cluster II and III of the current study might represent aspects of these latter motor competence profiles, which may display a different developmental and learning process in movement contexts compared to children in cluster I. The utility of classifying children’s motor competence level based upon the structure of their whole-body motor variability as they freely explore various interacting constraints, however, needs to be further evaluated against various task and individual constraints. As a limitation of the current study that warrants further investigation, group organisation based upon entropy needs to be compared with existing standardized assessment batteries and other potential evaluations of children’s motor level. Furthermore, longitudinal studies with multiple timepoints can provide important insights into the developmental process associated with the structure of movement variability and the impact of, e.g., level and type of sports/physical activity practice by children, as well as other individual constraints that might impact upon motor competence (Barnett et al., 2016).

## 5. Conclusion

In our study, based on a modest sample size, a hierarchical clustering analysis of sample entropy obtained from multiple joints throughout the body revealed three clusters of children 7–10 years old with distinct profiles. Those clusters are heterogeneous in terms of the repeatability of joint movements in combination with the level of spatiotemporal exploration, a dynamic that could not be explained by between-cluster differences in age and gender distributions. Those findings suggest that providing children with opportunities for motor action via free exploration and discovery, as embedded in the balance beam task on the VR playground used in our study, can lead to the identification of specific profiles and individual differences in the nonlinear structure of movement variability, which can aid in further understanding and developing assessments for children’s gross motor competence.

## Data availability statement

The raw data supporting the conclusions of this article will be made available by the authors, without undue reservation.

## Ethics statement

The studies involving humans were approved by Norwegian Social Science Data Services (NSD), part of the Norwegian Agency for Shared Services in Education and Research. The studies were conducted in accordance with the local legislation and institutional requirements. Written informed consent for participation in this study was provided by the participants’ legal guardians/next of kin.

## Author contributions

HL, ES, OS, and LS contributed to designing the research and collecting the data. HL analysed the data and wrote the first draft of the manuscript. All authors gave feedback and contributed to the manuscript and approved the submitted version.
